# Periostin: An Emerging Molecule With a Potential Role in Spinal Degenerative Diseases

**DOI:** 10.3389/fmed.2021.694800

**Published:** 2021-08-27

**Authors:** Daxue Zhu, Wupin Zhou, Zhen Wang, Yidian Wang, Mingqiang Liu, Guangzhi Zhang, Xudong Guo, Xuewen Kang

**Affiliations:** ^1^Lanzhou University Second Hospital, Lanzhou, China; ^2^Orthopaedics Key Laboratory of Gansu Province, Lanzhou, China; ^3^The 947th Army Hospital of the Chinese PLA, Kashgar, China; ^4^People's Hospital of Ningxia Hui Autonomous Region, Yinchuan, China

**Keywords:** periostin, osteoarthritis, extracellular matrix, spinal degeneration, signaling pathways

## Abstract

Periostin, an extracellular matrix protein, is widely expressed in a variety of tissues and cells. It has many biological functions and is related to many diseases: for example, it promotes cell proliferation and differentiation in osteoblasts, which are closely related to osteoporosis, and mediates cell senescence and apoptosis in chondrocytes, which are involved in osteoarthritis. Furthermore, it also plays an important role in mediating inflammation and reconstruction during bronchial asthma, as well as in promoting bone development, reconstruction, repair, and strength. Therefore, periostin has been explored as a potential biomarker for various diseases. Recently, periostin has also been found to be expressed in intervertebral disc cells as a component of the intervertebral extracellular matrix, and to play a crucial role in the maintenance and degeneration of intervertebral discs. This article reviews the biological role of periostin in bone marrow-derived mesenchymal stem cells, osteoblasts, osteoclasts, chondrocytes, and annulus fibrosus and nucleus pulposus cells, which are closely related to spinal degenerative diseases. The study of its pathophysiological effects is of great significance for the diagnosis and treatment of spinal degeneration, although additional studies are needed.

## Introduction

Each vertebra of the spine includes two parts: an intervertebral disc (IVD) and a bony vertebral body. The IVD consists of a central jelly-like nucleus pulposus (NP), a rigid annulus fibrous (AF), and upper and lower cartilaginous endplates, which connect the upper and lower vertebral bodies and withstand conditions such as compression, extension, bending, and torsion, and thus play an important role in bearing weight and cushioning pressure loads (1, 2). Spinal degeneration is a common clinical disease that includes vertebral body and intervertebral disc degeneration (IVDD). Disc degeneration is a common and important form of degeneration (3, 4). Long-term low back pain is a common clinical manifestation of chronic disease (1, 5). According to the Global Burden of Disease Study 2017, low back pain is a common condition that can affect up to 80% of the global population (6). It not only affects the life and work of patients but also causes heavy economic burdens on patients, their families, and society.

In recent years, the role of the extracellular matrix (ECM) in modulating the activity of tissue cells has been extensively studied. ECM proteins act not only as scaffolds for the organization of cells, but also as multifunctional regulators of cell behavior. For example, ECM proteins can modulate the activity or bioavailability of extracellular signaling molecules, such as growth factors, cytokines, chemokines, and extracellular enzymes, or directly bind to signals through cell surface receptors to regulate cell functions (7, 8). In addition, it is increasingly acknowledged that ECM proteins can alter local endogenous signal transduction responses to growth factors and cytokines (9). Therefore, ECM proteins have potential as biomarkers and precise molecular targets for therapeutic intervention in many chronic tissue diseases and even tumors (10, 11). Similar to most other tissues, IVD cells also rely on synergistic synthesis, maintenance, and degradation with the ECM to regulate and maintain the health of the IVD (12, 13).

Periostin (POSTN), an ECM protein, is the first adhesion molecule identified in the mouse osteoblast cell line MC3T3-E1, and is also called osteoblast-specific factor 2 (OSF-2). It is closely homologous to the insect cell adhesion molecule Fasciclin 1 (14). Although POSTN is not directly involved in the formation of the ECM, it plays a dynamic role in mediating the communication between cells and the surrounding microenvironment, and usually triggers specific effects (15). For example, POSTN can participate in ECM remodeling by interacting with collagen, fibronectin, tenascin C, and POSTN itself in a multi-affinity homotypic manner (16, 17). According to published reports, POSTN is also expressed in intervertebral cells, mesenchymal stem cells (MSCs), and osteoblasts, and plays a vital role in regulating bone density and IVD homeostasis (18, 19).

In short, the role of POSTN in affecting IVD cell function and vertebral body mass and density has been explored *in vivo* and *in vitro*. POSTN expression has been shown to be related to spinal degeneration and to play an important role in the activation and progression of related cell differentiation and pathology. Therefore, understanding its functions and signaling properties is essential to improve our knowledge of healthy cell–matrix interactions, although it is clear that more studies are needed to elucidate this.

## Structure and Function of POSTN

POSTN was originally identified as OSF-2. Later, because it is preferentially expressed in the periosteum and periodontal ligaments and plays a key regulatory role in the formation and maintenance of bones and teeth, it was renamed periostin by Takeshita et al. (20) and Horiuchi et al. (21). POSTN is an ECM protein with a molecular weight of 90 kDa. Its protein structure consists of an amino-terminal EMI domain, a tandem repeat of four FAS1 domains, and a carboxy-terminal domain (CTD) (22). These distinct domains have been demonstrated to bind to many proteins, including ECM proteins, and have different biological functions (23). It is precisely because of this special multi-domain structure that POSTN can help ECM proteins assemble into highly complex extracellular network structures. Therefore, the multi-domain structure of POSTN serves as the basis for its role in scaffolding in the ECM environment (24).

**Figure 1:**
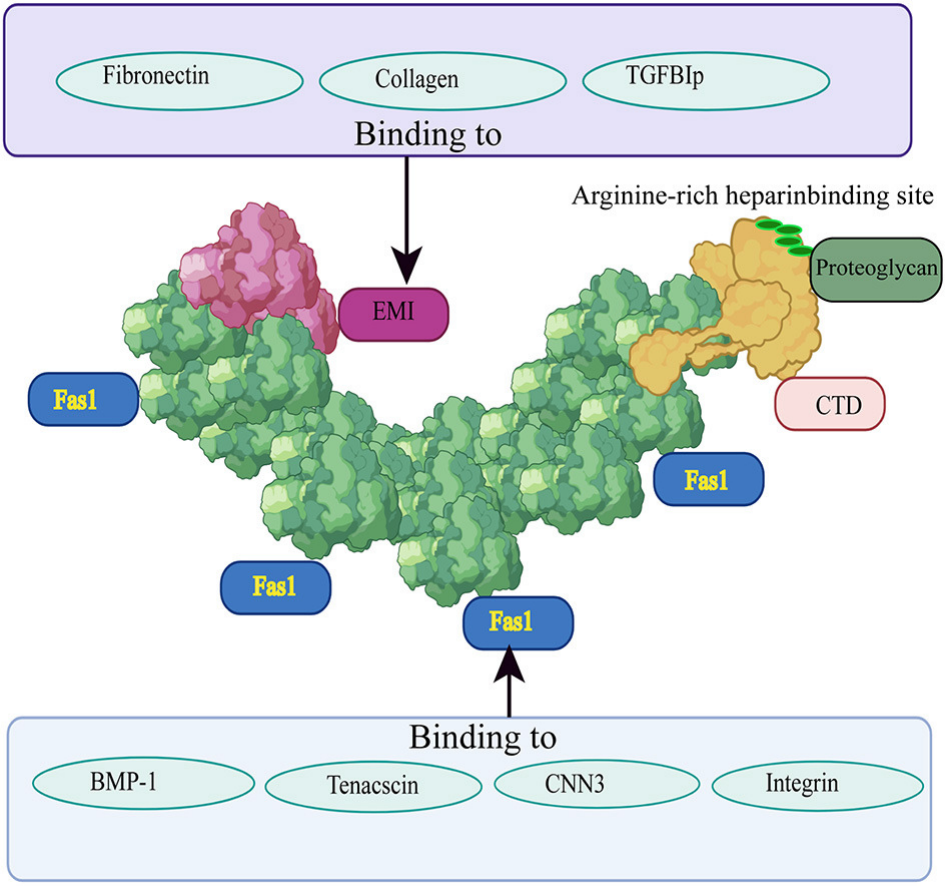
Structure of POSTN and its interactions with other ECM proteins. The EMI domain is depicted in red, the four-repeat FAS1 domain is shown in green, and the CTD is highlighted in yellow. Fibronectin, collagen II, and TGFB1 can bind to the EMI domain; the four repeated FAS1 domains can interact with BMP-1, tenascin C, CCN3, and integrin; the CTD includes an arginine-rich heparin binding site which is responsible for binding to proteoglycans. Different regions bind to different proteins and play different roles.

The EMI domain, belonging to the EMILIN family, consists of approximately 80 amino acid residues, including six highly conserved cysteine residues (25). It can bind to collagen types I, II, and IV (26) and to fibronectin (27), and promote the infiltration and contraction of the ECM (22, 28). Moreover, the EMI domain is essential for POSTN multimerization, and promotes collagen cross-linking by forming a network structure with fibronectin and tenascin C (29). The FAS1 domain is composed of four repeated FAS1 structural sequences in tandem, and was originally found in Fasciclin 1, a nerve cell adhesion molecule of Drosophila (30). The four tandem repeats of FAS1 are thought to exist only in TGFB1 (βigh3), stabilins, and POSTN in humans (31, 32). The FAS1 domain is a member of the Fasciclin 1 family and can bind to tenascin C, bone morphogenetic protein 1 (BMP-1), and cellular communication network factor 3 (CCN3), a member of the CCN family (33). The function of the FAS1 domain is to act as an anchor point to mediate the interaction of the EMI domain with other ECM components. The CTD includes an arginine-rich heparin binding site, which makes it possible to purify POSTN using heparin columns (34). Different isomers of POSTN, all having cell adhesion functions, can be formed according to the extent of C-terminal shearing (35). The heparin binding site is responsible for binding to proteoglycans (36, 37). It plays an important role in regulating cell processes such as cell migration and growth factor signal transduction by interacting with heparan sulfate proteoglycans on the cell surface (38). The structure of POSTN and a schematic diagram of its different domains interacting with other ECM proteins are shown in Figure 1.

## POSTN is Involved in Regulating Vertebral Body Bone Mass and Density

Spinal degeneration includes bony vertebral body and IVD degeneration, which is not only related to the physiological process of aging (39), but can also be promoted by many other factors, such as mechanical pressure, inflammatory factors, and oxidative stress (40), resulting in an increased risk of spinal imbalance, osteoporosis, and even degenerative fractures (41). Among the factors affecting vertebral degeneration, osteogenic differentiation of bone marrow-derived mesenchymal stem cells (BMSCs) and the regulation of the distribution of osteoblasts and osteoclasts are the most important (42).

BMSCs are known as key factors in the strategy of bone tissue engineering because of their self-renewal and multi-differentiation abilities (43). It is well-known that bone formation or ossification occurs through intramembranous or intrachondral ossification, and intramembranous ossification occurs via differentiation of BMSCs into osteocytes (44). In addition, BMSCs in the vertebral body can differentiate into osteoblasts and adipocytes. When the ability of BMSCs to differentiate into osteoblasts is weakened, while their ability to differentiate into adipocytes is enhanced, adipose tissue accumulates in the vertebral body, while the bone mass, density, and stiffness of the vertebral body are reduced. Therefore, osteogenic differentiation of BMSCs is necessary for the maintenance of normal bone homeostasis and bone defect repair (45, 46).

Osteoblasts are critical components of the bone multicellular unit and play an important role in bone remodeling, which is essential for maintaining the structural integrity and metabolic capacity of the skeleton (47). The accumulation of osteoclasts increases the osteoclast effect, which promotes bone metabolism and reduces bone formation, and ultimately affects the bone mass and density of the vertebral body (48, 49). In the elderly, vertebral body degeneration, for instance during osteoporosis or upon osteoporotic fractures, is caused by the deregulation of bone metabolism due to the imbalance of osteoblasts and osteoclasts. Therefore, as one of the main manifestations of spinal degeneration, bony vertebral body degeneration is closely related to the direction of BMSC differentiation and the balance between osteoblasts and osteoclasts (50–52). In the following sections we describe the effect of POSTN on the determination of vertebral body bone mass and density through its activity in BMSCs, osteoblasts, osteoclasts, and osteocytes.

### POSTN Induces Osteogenic Differentiation of BMSCs

By analyzing the secretome of MSCs via proteomics methods combined with two-dimensional gel electrophoresis and tandem mass spectrometry, Coutu et al. showed for the first time that POSTN is highly expressed in MSCs and plays a key role in the mineralization of the ECM (53). This conclusion was confirmed by another study. Interestingly, the authors of the latter concluded that POSTN not only is secreted from BMSCs, but also supports tendon formation when overexpressed (54). In addition, POSTN can also bind to integrin α6β4, which is very important for the survival and differentiation of BMSCs (55).

These findings have prompted several studies on the mechanism of POSTN-mediated BMSC differentiation. For example, Zhang et al. (56) examined POSTN expression in three groups of human BMSCs: wild-type MSCs, erythropoietin-producing hepatocyte B4 (EPHB4)-overexpressing MSCs, and MSCs with siRNA-mediated inhibition of EPHB4 expression. After stimulation with ephrin B2-FC, POSTN expression was significantly increased in the first two groups, but not in the third. EPHB4 is a receptor expressed in MSCs/pre-osteoblasts and can interact with ephrin B2 to forward signals that promote osteogenic differentiation (57). This finding suggests that POSTN is a downstream mediator of EPHB4 implied in the induction of the osteogenic differentiation of BMSCs. Moreover, these authors studied the effects of POSTN and ephrin B2-FC on the osteogenic differentiation of BMSCs by measuring the level of the osteogenic marker alkaline phosphatase (ALP) and counting bone nodules, and regarded BMSCs with stimulated POSTN expression and blocked integrin αvβ3 expression as positive and negative controls, respectively. The results showed that both ephrin B2-FC and POSTN could induce osteogenic differentiation of BMSCs. Finally, these authors also studied the osteogenic differentiation mechanism mediated by POSTN and EPHB4. The results showed that both POSTN and EPHB4 could increase the expression of p-GSK3-β (Ser9) and β-catenin, as well as that of the osteogenic markers type I collagen, RUNX2, and ALP, but not in the EPHB4 siRNA-treated group and the negative control group. These results indicate that the expression of POSTN induced by the EPHB4 signaling pathway downregulates GSK-3-β (Ser9) activity through the Wnt pathway to promote the osteogenic differentiation of BMSCs (56).

In a subsequent study, the effect of POSTN on the osteogenic differentiation of MSCs bearing a modified cytotoxic T-lymphocyte associated protein 4 (MSCs-CTLA4) was examined by establishing a MSCs-CTLA4 model, because previous studies found that MSCs-CTLA4-based xenografts had better osteogenic ability than MSC-based xenografts (58). Immunohistochemical analysis showed that POSTN was expressed in both MSCs and MSCs-CTLA4 under tissue-engineered bone and immune-activated conditions, but showed higher expression in MSCs-CTLA4; moreover, collagen type I expression was consistent with this pattern. Further confirming the role of POSTN in inducing osteogenesis in MSCs-CTLA4 cells, the expression of osteogenic markers, including RUNX family transcription factor 2 (RUNX2), collagen type I, osteocalcin, osterix, and ALP, as well as the formation of calcium nodules, in MSCs increased after treatment with soluble POSTN. However, when soluble POSTN was neutralized, these effects were diminished (59). These results indicate that CTLA4 can promote ectopic osteogenesis by maintaining POSTN expression in xenotransplants. However, the specific mechanism of action remained unclear until another study demonstrated that CTLA4 maintains POSTN expression by regulating the phosphorylation of LDL receptor related protein 6 (LRP6) and activating the Wnt/β-catenin signaling pathway under immune activation (60).

**Figure 2:**
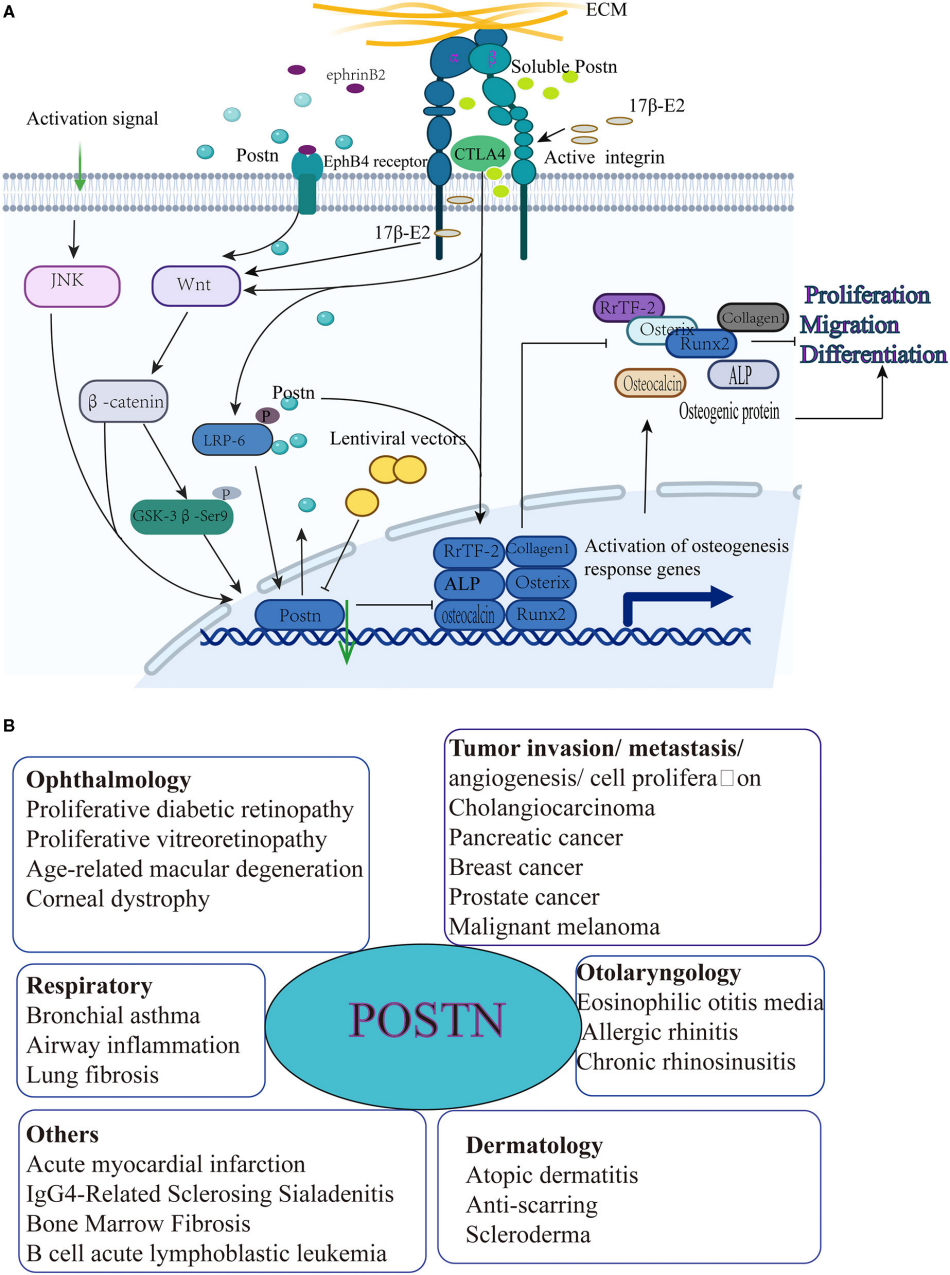
**(A)** POSTN induces osteogenic differentiation, migration, and proliferation of MSCs. (1) The receptor erythropoietin-producing hepatocyte B4 (EPHB4) interacts with ephrin B2, thereby inducing POSTN expression, which in turn promotes the expression of p-GSK-3-β (Ser9); this leads to the downregulation of GSK-3-β (Ser9) activity through the Wnt pathway to increase collagen type I, RUNX2, and ALP expression in order to promote the osteogenic differentiation of BMSCs. (2) CTLA4 maintains POSTN expression by regulating the phosphorylation of LDL receptor related protein 6 (LRP6) and activating the Wnt/β-catenin signaling pathway under immune activation conditions to promote RUNX family transcription factor 2 (RUNX2), collagen I, osteocalcin, osterix, and ALP expression, as well as calcium nodule formation in BMSCs. (3) The expression of osteogenic markers in MSCs increases after treatment with soluble POSTN. However, when soluble POSTN is neutralized, these effects are diminished. (4) 17β-estradiol (17β-E2) induces osteogenic differentiation of BMSCs in ovariectomized rats through the POSTN-Wnt/β-catenin pathway to promote osteogenic effects. **(B)** Periostin and associated diseases [adapted from (23)].

In addition, when exploring the role of POSTN in the regulation of bone formation by 17β-estradiol (17β-E2), Li et al. (61) found that the expression of POSTN in BMSCs from mice subjected to ovariectomy and treated with 17β-E2 was higher than that in BMSCs from mice belonging to the sham and ovariectomy only groups. In order to study the role of POSTN in the regulation of bone formation by 17β-E2, they overexpressed POSTN by transfecting POSTN-GFP lentivirus. And RT-PCR and ALP staining were performed 7 days after osteogenic induction to investigate the osteoblastic differentiation. The results showed that the expressions of POSTN, Wnt3a and β-catenin in the Lev-POSTN group were higher than those in the control group. The overexpression of POSTN partially reversed the levels of RUNX2 and ALP, and partially restored the ALP activity of OVX-BMSCs. Then, they also treated OVX-BMSCs with *Postn*-siRNA to reduce the expression of POSTN in order to further evaluate the relationship between POSTN and Wnt/β-catenin signaling pathway under 17β-E2 stimulation. The expression of POSTN, Wnt3a and β-catenin was measured by Western blotting. The results showed that the expression of POSTN, Wnt3a and β-catenin protein in the *Postn*-siRNA group was lower than that of the negative control siRNA group. Finally, it was concluded that 17β-E2 can induce osteogenic differentiation of BMSCs in ovariectomized rats through the POSTN-Wnt/β-catenin pathway to promote osteogenic effects and reduce osteoporosis. POSTN may be a new target gene for anti-osteoporosis treatment, and, due to its wide expression profile, its role in the regulation of stem cell function is emerging (62). The mechanism by which POSTN regulates the osteogenic differentiation of BMSCs is shown in Figure 2A.

Apart from BMSCs, POSTN also acts in other types of stem cells. For example, POSTN interacts with integrin αv in hematopoietic stem cells (HSCs) to regulate HSC homeostasis in the bone marrow niche (63). POSTN has also been reported to promote the migration and proliferation potential of periodontal ligament mesenchymal stem cells (PDLSCs) via the JNK signaling pathway, revealing the underlying mechanism by which POSTN enhances the function of MSCs (64). POSTN is also expressed in neural stem cells (NSCs), and the interaction between different niches that POSTN mediates is crucial in the adult neurogenic niche (65). Similar to its role in other adult stem cells, POSTN can participate in the regulation of NSC function through integrin signals and promote the proliferation and differentiation of NSCs. Moreover, POSTN supports the regeneration potential of neurons through the maintenance or activation of stem cells (66). Cancer stem cells (CSCs) have been shown to express POSTN via either autocrine or paracrine pathways; this process plays an important role in the functional maintenance of CSCs. For example, POSTN secretion increases the invasiveness of CSCs and promotes metastasis (67). In many cancer models, the expression of POSTN and its receptors also plays a crucial role in tumorigenesis (68).

In general, POSTN is considered to be a key molecule that plays a promoting role in the proliferation, migration, and osteogenic differentiation of MSCs, although these effects may be modulated through the regulation of autocrine or paracrine pathways (64, 69); moreover, POSTN indirectly affects the bone density and quality of the vertebral body, and ultimately participates in the establishment and progression of IVDD. In addition to IVDD, POSTN plays an extremely important role in various other systemic diseases, such as asthma fibrosis, infarcted myocardial scar formation, and tumorigenesis (70). In a recent study, POSTN was shown to promote the progression of B-cell acute lymphoblastic leukemia (B-ALL) by regulating the expression of monocyte chemoattractant protein 1 (MCP-1 or CCL2) in leukemia cells (71). The mechanisms by which POSTN participates in its related diseases are shown in Figure 2B.

### Activity of POSTN in Osteoblasts and Osteoclasts

POSTN was originally identified in MC3T3-E1 osteoblasts and was found to play a role in mediating transforming signal response to growth factor-β (TGF-β) and cell adhesion (21). Later, Litvin et al. isolated an isoform of POSTN from osteoclasts called periostin-like factor and found that this played an important role in osteoblast differentiation (72). A recent study analyzed the effects of POSTN on the differentiation of osteoblasts by examining three osteoblast models isolated from the mouse periosteum, calvaria, and MC3T3-E1 cell line. The results showed that Postn was expressed in all three models during the whole differentiation process in a time-dependent manner. In the differentiation process of the calvaria and MC3T3-E1 models, the expression of Postn first appeared on the third day, while resulting detectable only on the seventh day in the mouse periosteum model; furthermore, its expression increased by 3–10 times between the sixth and the 14th day, when it reached its peak. After differentiation was complete and mineralization begun, the expression of Postn rapidly declined (73). Subsequent studies also confirmed that POSTN secreted by osteoblasts participates in the early stages of bone development and bone formation by promoting osteoblast differentiation and survival, and is a key regulator of bone microarchitecture, strength, and mass (55, 74). However, the expression of the osteoblast differentiation markers collagen type I, osteocalcin, osteopontin, ALP, and RUNX2 was decreased in an osteoblast model with Postn knockout. This indicates that inhibition of Postn can suppress cell proliferation and differentiation, resulting in decreased bone mass and strength, decreased collagen cross-linking and demineralization, and increased micro-damage (75).

Nevertheless, the mechanism by which POSTN induces osteoblast differentiation was unknown until Zhang et al. (76) studied the expression of Postn and semaphorin 3A in osteoblasts under mechanical stress *in vitro*. Semaphorin 3A is an axon-guiding molecule secreted by osteoblasts and osteocytes, and can enhance bone formation, increase bone volume, and accelerate bone regeneration by inhibiting bone resorption (77, 78). It was found that the gene expression of Postn and semaphorin 3A in MC3T3-E1 cells was increased under mechanical stimulation. When an MC3T3-E1 cell culture was treated with recombinant POSTN, the level of semaphorin 3A in its supernatant was significantly increased in a dose-dependent manner, promoting the activation and differentiation of osteoblasts through the Wnt/β-catenin signaling pathway. Moreover, previous studies have confirmed that high levels of mechanical stretch can induce osteoclast apoptosis (79, 80), but the intensity of the apoptosis signal is weakened after POSTN preconditioning. A subsequent study revealed that POSTN expression increased in osteoblast-like MG-63 cells subjected to mechanical stretch through the activation of the TGF-β signaling pathway (81). This finding suggests that POSTN protects osteoblasts from mechanical stretch-induced apoptosis (81).

In the same year, Meng et al. found that apoptosis of osteoblasts was induced by melatonin through the activation of the endoplasmic reticulum stress (ERS)-associated eukaryotic initiation factor 2α (eIF2α)-activating transcription factor 4 (ATF4) pathway; subsequently, the cascade effects of ccAAT/enhanced binding protein homologous protein (CHOP), caspase 3, and c-Jun N-terminal kinase (JNK) were triggered to promote apoptosis. Similarly, overexpressed POSTN can act as a protective factor for osteoblasts because it can hamper melatonin-induced apoptosis by inhibiting the eIF2α-ATF4 pathway through the suppression of the protein kinase R-like endoplasmic reticulum kinase (PERK) pathway (82).

**Figure 3:**
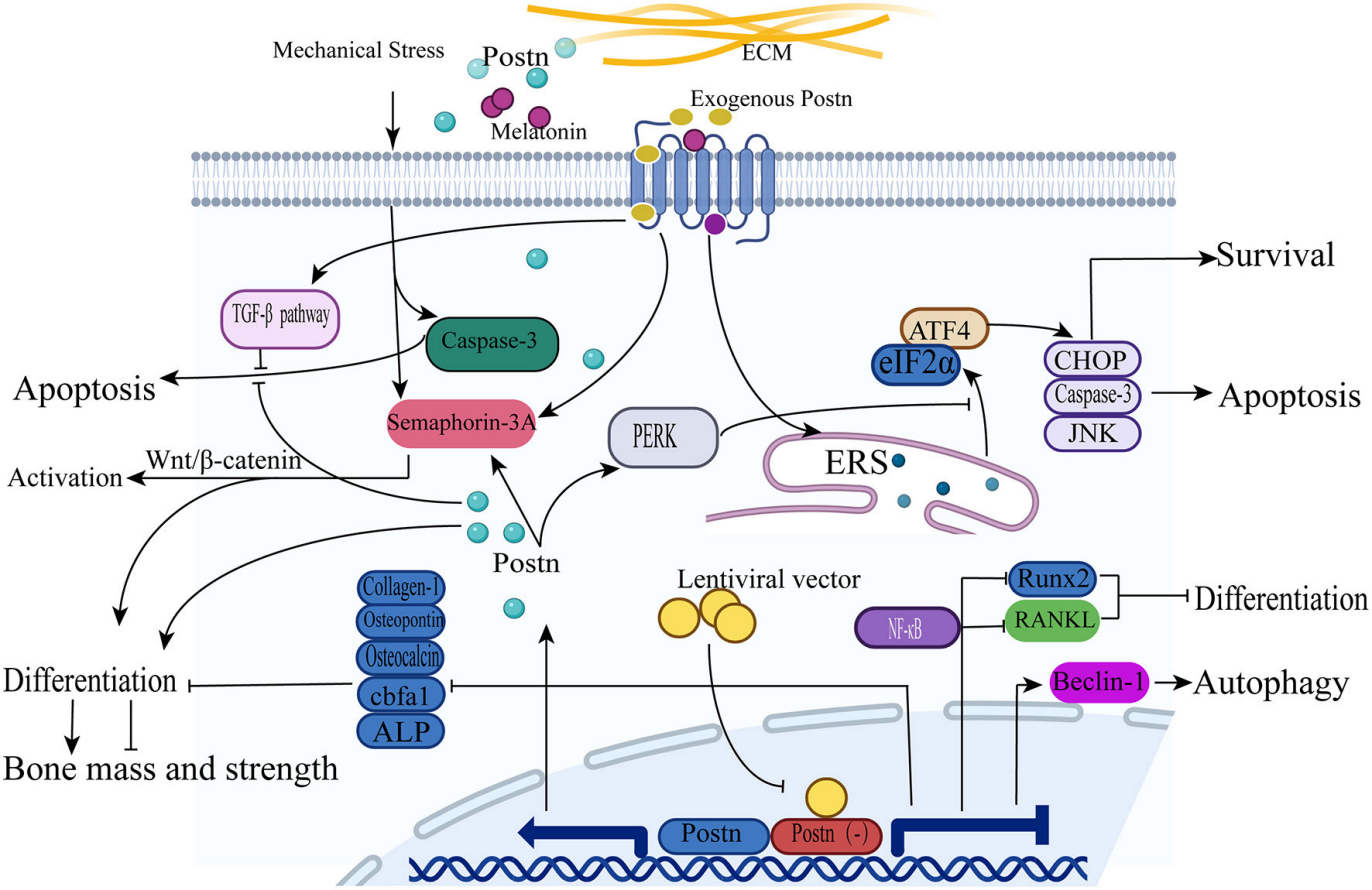
POSTN induces osteoblast differentiation, survival, and apoptosis. (1) Upon treatment with recombinant POSTN, the expression of semaphorin 3A is increased and promotes the activation and differentiation of osteoblasts through the Wnt/β-catenin signaling pathway. (2) The intensity of osteoclast apoptosis signals is weakened by POSTN expression through the TGF-β signaling pathway under a high level of mechanical stress. (3) The expression of the autophagy-related protein beclin 1 in osteoblasts is increased after Postn silencing. (4) The expression of the proteins RUNX2 and RANKL is significantly reduced after Postn gene silencing through the activation of the NF-κB signaling pathway. (5) Osteoblast apoptosis is induced by melatonin through the activation of the ERS-eIF2α-ATF4 pathway; subsequently, the cascade effects of CHOP, caspase 3, and JNK are triggered to promote apoptosis. POSTN overexpression can inhibit the eIF2α-ATF4 pathway by suppressing the PERK pathway to protect osteoblasts from apoptosis.

In addition, Qin et al. constructed a postn knockout (KO) model in MC3T3-E1 osteoblasts using a lentiviral vector to study the effect of Postn inhibition on autophagy in osteoblasts. The results showed that the expression of the autophagy-related protein beclin 1 in osteoblasts was increased, suggesting that the autophagy function of osteoblasts was enhanced. This also indicates that POSTN inhibits autophagy in osteoblasts, although the specific signaling pathway mediating this effect has not yet been identified (83). In a subsequent study, these authors demonstrated that the protein expression of RUNX2 and RANKL was significantly reduced after Postn gene silencing, and speculated that this process was achieved through the NF-κB signaling pathway (84). The mechanisms of action of POSTN in osteoblasts are shown in Figure 3.

According to the literature, there appears to be no relationship between POSTN expression and osteoclasts (75, 85). In fact, although cortical bone remodeling was increased in postn KO mice at 1 year of age, there was no change in osteoclast activity and number in long bones of postn KO mice (86). However, subsequent studies have redefined the relationship between POSTN and osteoclasts, and Postn has been shown to be expressed at low levels in mouse long-bone osteoclasts differentiated *in vitro* (73). Moreover, the number and activity of wild-type osteoclasts co-cultured with postn KO mouse primary osteoblasts were higher than those of wild-type osteoblasts co-cultured with postn KO mouse primary osteoblasts. In turn, the addition of recombinant POSTN to osteoclast cultures reduced the number and activity of osteoclasts (87).

### POSTN Exerts an Anti-apoptotic Effect on Osteocytes and Affects Bone Metabolism

Although POSTN is not expressed in bone cells (88), the relationship between POSTN and bone cells has been demonstrated by several studies. For instance, POSTN has been shown to interact with Notch receptors and to exert an anti-apoptotic effect on osteocytes under mechanical stress (89). Similarly, intraperitoneal injection of a high dose of zoledronate sodium into wild-type and postn KO mice treated with a high dose of zoledronate for 3 months reduced the number of osteocytes in both groups, but the number of empty osteocyte lacunae increased in the latter; this result confirmed that POSTN exerts an anti-apoptotic effect in osteocytes (85). POSTN also plays a vital role in bone formation, and in determining its mass and density (90). The observation that the quality of cortical bone decreases due to the reduction of periosteal bone formation in a postn KO mouse model supports this view (55, 91). Another strong piece of evidence is that in mouse strains where Wnt16 gene KO leads to a decrease in cortical bone mass, the expression of POSTN in periosteal cells is reduced (92). POSTN expression also modulates bone homeostasis (93).

The mechanism by which POSTN influences bone formation, quality, metabolism, and density has been revealed in follow-up studies. For example, POSTN directly stimulates the Wnt signaling pathway and osteoblast function while inhibiting the expression of sclerostin (SOST) to mediate the anabolic response of the bone to parathyroid hormone. Therefore, defective POSTN expression impairs bone formation, as parathyroid hormone cannot effectively improve its cortical structure and strength. Moreover, POSTN expression may be involved in the remodeling of bone cells near the inner cortical ridge (91).

In addition, recent data indicated that cathepsin K, a protease involved in the process of bone resorption, may also modulate periosteal bone formation by regulating POSTN expression (94, 95). In this context, the drug odanacatib, a cathepsin K inhibitor, is effective in reducing osteoporotic fractures and stimulating the formation of periosteal bone in ovariectomized adult monkeys (96). In another study, the anti-osteoporosis drug teriparatide, an anabolic drug that promotes bone remodeling in patients with osteoporosis, was found to trigger the secretion of POSTN to promote bone formation and counteract osteoporosis. Moreover, after teriparatide treatment, the level of POSTN in the circulation of patients increased significantly (97). Finally, POSTN plays a different role in each stage of bone repair: in the early stage of repair, it activates periosteal stem cells; during the active period, it can improve the deposition of fracture callus cartilage and bone; in the final stage, it induces bone bridging and rebuilding of the stem cell pool in the periosteum (98, 99).

In summary, these studies effectively illustrated that POSTN is associated with bone mass, density, and metabolism as it indirectly affects osteocyte apoptosis, although it is not directly expressed in osteocytes. Moreover, POSTN is not only a regulator of bone formation and bone mineral density but also a potential biomarker for osteoporosis and fracture. It plays an irreplaceable role in the onset and development of vertebral degeneration, for instance during osteoporosis or upon osteoporotic fracture.

## The Activity of POSTN in IVD Cells

IVD cells can be distinguished into three main groups: NP cells, AF cells, and superior and inferior endplate chondrocytes. Altogether, these cells constitute the IVD, a flexible joint localized between adjacent spinal vertebrae (2, 100). The tough AF is the circular exterior of the IVD and surrounds the inner soft NP. Its function is to prevent the NP from herniating or leaking out of the disc by sealing the nucleus and distributing any pressure and force imposed on the IVD (101, 102). The elastic NP is mainly composed of water (70–90%), NP cells, proteoglycan, and type II collagen (3). When the spine moves, the spherical structure of the NP contributes significantly to dispersing pressure, supporting large-angle and high-frequency movement, and assisting other parts of the spine in the completion of physiological activities (103, 104). Finally, it has been suggested that the cartilage endplates absorb the small molecules and nutrients required for disc cells (39). In summary, the dysfunction of IVD cells causes loss of balance and reduces the effect of balancing pressure, leading to decreased function of the IVD and its eventual degeneration. In the following sections we describe the role of POSTN in each of the three above-mentioned IVD cell types, which mediates its indirect influence on the occurrence and development of IVDD.

### POSTN Induces Chondrocyte Apoptosis and Cartilage Degradation

Although the mechanisms involved in the binding of POSTN to the cartilage endplate of the IVD have not been analyzed in detail, studies on cartilage of patients with osteoarthritis (OA) have extensively reported that POSTN can interact with chondrocytes and enhance cell proliferation and survival, migration, and metastasis. For example, Chen et al. (105), while exploring the molecular differences between light and dark hypertrophic chondrocytes by qPCR, found that the most highly differentially expressed dark cell-specific gene was Postn. Other studies also detected a significant increase in the protein expression of POSTN between OA and normal chondrocytes by immunohistochemistry (106, 107). Moreover, the POSTN isoforms 1 and 8 have been reported to be significantly highly expressed in chondrocytes, whereas others are mainly expressed in anterior cruciate ligament remnants, suggesting that differential expression patterns of POSTN and its splice variants exist in various tissues and cell types (19). POSTN was also found to contribute to the maturation and shape retention of tissue-engineered cartilage by promoting conformational changes in collagen molecules during tissue engineering (108). This newly discovered effect of POSTN could be useful in clinical applications in the future. These findings demonstrated that POSTN is not only expressed in chondrocytes and synovial fluid (SF) from OA patients but may also be a potential biomarker and contributor to the progression of OA. Most importantly, POSTN also has potential value for applications in tissue repair and regeneration.

With the deepening of research, especially on the role of chondrocytes in OA, a large number of studies have revealed the role and mechanism of POSTN activity in chondrocytes. For example, a recent study reported that POSTN is upregulated in human and rodent OA chondrocytes. To promote OA, POSTN induces the expression of matrix metallopeptidase 13 (MMP13) and ADAMTS4 in a dose- and time-dependent manner. Moreover, MMP13 levels are regulated by endogenous POSTN, because when the Postn gene was inhibited by siRNA transfection, the levels of both endogenous POSTN and MMP13 in chondrocytes were reduced. This process was accomplished through the selective activation of Wnt/β-catenin signaling without the activation of the ERK1/2, p38, and NF-κB pathways (109). However, another study in the same year showed that the upregulation of IL-6, IL-8, MMP1, MMP3, MMP13, and nitric oxide synthase 2 (NOS2), which are induced by POSTN and known to be related to OA pathogenesis, was suppressed by NF-κB inactivation in chondrocytes. It has been suggested that POSTN triggers the activation of the NF-κB signaling pathway, denoted by the nuclear translocation of p65, and subsequently upregulates inflammatory factors and MMPs to induce chondrocyte apoptosis and accelerate cartilage degeneration (110). In human OA synovial cells, researchers not only detected higher gene and protein expression of POSTN than in normal cells, but also observed increased secretion of POSTN under the stimulation of the inflammatory cytokine IL-13, suggesting that both POSTN and IL-13 are involved in the pathological progression of OA. Mechanistically, IL-13 promotes the phosphorylation of signal transducer and activator of transcription 6 (STAT6) and activates STAT6 to upregulate POSTN expression. Moreover, POSTN expression was significantly inhibited by dexamethasone and leflunomide (111, 112). Although these results are inconsistent with the hypothesis of the selective activation of the Wnt/β-catenin signaling pathway, the results of POSTN overexpression in OA chondrocytes and SF are consistent with the previous hypothesis.

In addition, Chinzei et al. (113) studied the effects of anabolic (TGF-β1) and catabolic (IL-1β) factors on the expression of POSTN and found that the expression of POSTN did not change after treatment with either TGF-β1 or IL-1β. However, these factors affected chondrocyte metabolism. These authors also observed that exogenous POSTN treatment of chondrocytes stimulated the expression of MMP13, but did not alter Postn gene expression, while siRNA-mediated POSTN ablation inhibited expression of the POSTN and MMP13 genes, consistent with prior results (113). These findings suggest that exogenous POSTN can act as a catabolic factor promoting cartilage degradation. Subsequent studies revealed the following mechanism: POSTN-induced upregulation of MMP13 requires its interaction with and activation of discoidin domain receptor 1 (DDR1). DDR1 is a receptor tyrosine kinase that signals through the AKT-Wnt/β-catenin pathway to induce the degradation of collagen and proteoglycans in the cartilage (114).

**Figure 4:**
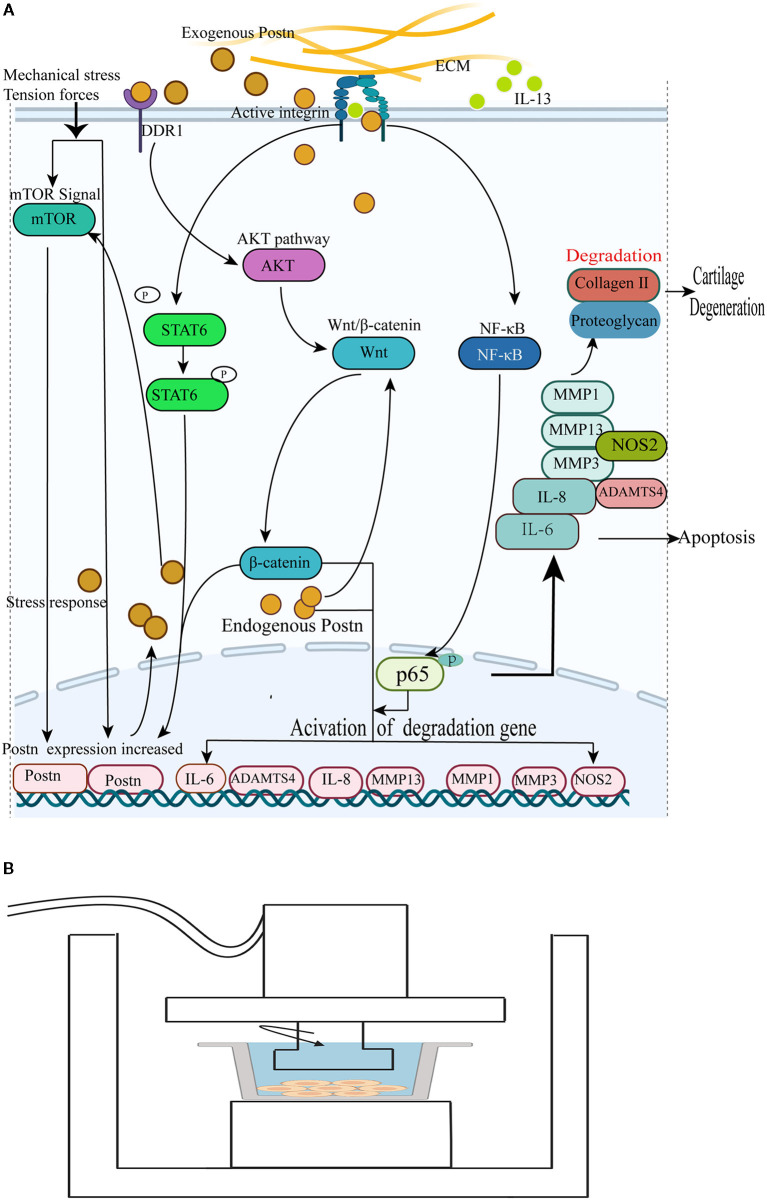
**(A)** POSTN promotes collagen and proteoglycan degradation to accelerate cartilage degeneration and loss. (1) Endogenous POSTN can promote the expression of MMP13 and ADAMTS4 by activating the canonical Wnt/β-catenin signal pathway to accelerate cartilage degeneration and chondrocyte apoptosis. (2) POSTN leads to the activation of the NF-κB signaling pathway, denoted by the nuclear translocation of p65, and subsequently upregulates IL-6, IL-8, MMP1, MMP3, MMP13, and NOS2 to induce chondrocyte apoptosis and accelerate cartilage degeneration. (3) IL-13 promotes the phosphorylation of STAT6 and activates STAT6 to upregulate POSTN expression, contributing to the pathological progression of OA. (4) Exogenous POSTN-induced upregulation of MMP13 requires its interaction with and activation of DDR1 to induce the degradation of collagen and proteoglycans in the cartilage through the AKT-Wnt/β-catenin pathway. (5) Mechanical stress can induce POSTN expression to increase chondrocyte apoptosis. The expression of POSTN increases in response to tendon forces via the mTOR pathway; in turn, POSTN enhances mTOR signals. **(B)** Schematic diagram of a mechanical stress induction device. The arrow indicates the direction of the mechanical force generated by the device. The gray line represents a culture dish with a monolayer of nucleus pulposus cells (115).

In summary, POSTN, previously reported as an osteoblast-specific factor, has been shown to be upregulated in both human and rodent OA chondrocytes. In addition, it is a catabolic factor promoting collagen and proteoglycan degradation to accelerate the pathogenesis of OA, and may act as a novel therapeutic target to prevent OA progression. The mechanisms of POSTN-mediated chondrocyte apoptosis and ECM degradation leading to cartilage degeneration and promotion of OA are shown in Figure 4A.

### Expression of POSTN in Annulus Fibrous and Nucleus Pulposus Cells

POSTN is predominantly expressed in collagen-rich fibrous connective tissues that are subjected to constant mechanical stress (17). Because of this characteristic, one could speculate that POSTN is also expressed in the IVD, especially in AF and NP cells. As expected, through the immunolocalization of POSTN in 11 specimens of surgical or donor lumbar IVD tissues, a study revealed that POSTN exists in the cytoplasm of some cells in the human inner and outer rings, as well as in the NP (18). The greatest proportion of POSTN-expressing cells was present in the outer annulus (88.8%), while the inner annulus tissue and the NP contained an average of 61.4 and 18.5% POSTN-expressing cells, respectively. Similar cellular patterns of POSTN localization have been observed in lumbar discs from sand rats (18). Moreover, additional studies have shown that the percentage of POSTN-positive cells in the inner ring is significantly negatively correlated with the age of the subjects (7, 18). However, a later study demonstrated that there is a positive relationship between POSTN expression and NP cell degeneration. Its authors measured POSTN gene expression in degenerative and non-degenerative NP cells by PCR, and the results showed that the mRNA and protein levels of POSTN increased in the degeneration group. Histological examination showed that, compared with non-degenerative IVD, human IVD displayed more fibrosis together with structural disorders and fragments. Finally, POSTN positive staining increased in human tissues and degenerated IVD of rat tails subjected to acupuncture (115).

Recently, using microarray and bioinformatics analyses, abnormal gene expression during aging of rat NP cells has been observed; among downregulated genes, Postn was the most significantly altered, a result confirmed by real-time PCR. This finding contradicts the results of previous studies showing that the mRNA and protein level of POSTN in IVD cells increases during IVDD (115, 116). The authors explained such discrepancy by discussing that the acupuncture mouse model represents a rapid damage of the IVD, which stimulates the expression of POSTN to regulate the structure of the ECM. However, the model in their experiment was natural aging, which is more similar to the process of NP degradation. Consistent with these results, Graja et al. (117) reported that POSTN loss in aging adipose tissue is closely related to its age-related ECM changes. In addition, bioinformatics analysis revealed that the KEGG pathways that were enriched with the highest score in the gene module relative to the NP cell aging process were “ECM–receptor interaction,” “local adhesion,” and “PI3K-Akt signaling pathway”; however, more experiments are needed to confirm these results (116, 118).

In summary, these studies have demonstrated the relationship between POSTN expression and IVDD, and the potential mechanism of NP cell senescence has been studied on a genome-wide scale. This suggests that POSTN may be a promising new prognostic marker and therapeutic target, and further studies are needed to expand our understanding of its role in IVDD. However, it is regrettable that, despite reports that POSTN is highly expressed in the AF, there is a lack of further literature on this topic.

## The Role of POSTN in the Maintenance of the Extracellular Matrix

In addition to the well-known NP cells, annulus fibroblasts, and endplate chondrocytes, the IVD also includes ECM components. The ECM is a mixture of molecules secreted by cells and provides biochemical and structural support for cells, tissues, and organs (11). A large number of existing studies have demonstrated that the occurrence and development of IVDD is closely related to excessive ECM catabolism. An increasing amount of direct genetic evidence links the development, maintenance, and repair of the IVD with the coordinated interaction of the ECM components in the respective IVD niches (119). Therefore, to delay the progression of IVDD, it is necessary not only to regulate the function of NP cells but also to restore the metabolic balance of the ECM (120). Thus, it has been suggested that the ECM may be a new biomarker for the treatment of IVDD or a major candidate target for treatment.

POSTN is an ECM protein composed of multiple domains, as described previously (20). It has different physiological functions in tissues when combined with other ECM molecules. It has been proven that POSTN can not only serve as the basis of complex ECM architecture, but also be used as a scaffold for assembling ECM and accessory proteins through its multi-domain structure (29, 121, 122). These distinct domains have been shown to bind to many proteins, including ECM proteins (collagen types I and V, fibronectin, tenascin, and laminin), matricellular proteins (CCN3 and βig-h3), and enzymes that catalyze covalent cross-linking between ECM proteins (lysyl oxidase and BMP-1) (9, 24). For example, Kii et al. (27) observed that the proximal end of POSTN binding to fibronectin is located in the endoplasmic reticulum of fibroblasts. Moreover, POSTN can enhance the secretion of fibronectin from the endoplasmic reticulum to the extracellular environment. It was revealed that POSTN interacts directly with fibronectin before secretion and plays an important role in fibronectin secretion.

POSTN has also been found to interact with collagen and form a complex with type I collagen. It can also directly bind to type V collagen and plays an important role in the formation of collagen fibers (16, 17). Although the binding site of collagen has not been determined, the process of collagen fiber formation is important for tissue repair and IVDD. In this process, the interaction of POSTN with fibronectin is a key factor (123). Moreover, tenascin C interacts with and binds to the FAS1 domain of POSTN. Tenascin C is an ECM protein that can form six-arm oligomers by multimerization through disulfide bonds at its amino terminus (29, 124). In the process of collagen production, POSTN and intracellular tenascin C form a network structure that combines with fibronectin to form a cross-linked type I collagen scaffold (9).

In addition, POSTN interacts with BMP-1, a pro-collagen processing enzyme that removes the carboxy-terminal propeptide of several proteins (125). Experiments by Maruhashi and Hwang et al. showed that POSTN directly binds to and interacts with BMP-1 (28, 126). The result of this interaction is the enhanced proteolytic activation of lysine oxidase (LOX), an enzyme that catalyzes the intermolecular cross-linking of collagen molecules and is essential for the formation of collagen fibers (127). Interestingly, LOX can also be connected to the EMI domain of POSTN via fibronectin (126, 128).

Finally, Takayama et al. (33) proved that POSTN can interact with CCN3 to enhance its deposition in the ECM. CCN3 includes four domains: an insulin-like growth factor-binding protein-like domain (IGFBP), a von Willebrand type C-like domain (VWC), a thrombospondin type 1-like domain (TSP1), and a carboxy-terminal domain (CT). In this assembly process, the TSP1 and CT domains of CCN3 interact with the EMI domain of POSTN.

In addition to the proteins described above, POSTN has been reported to interact with members of the integrin superfamily. Although direct evidence of the interaction between POSTN and integrins has not been provided, POSTN is believed to interact with integrins, including αVβ3, αVβ5, and α6β4, that promote cell proliferation, cell migration, epithelial-to-mesenchymal transformation, and modulation of the biomechanical properties of connective tissues (63, 129, 130).

In conclusion, POSTN interacts with multiple ECM proteins, as shown in Table 1 and Figure 1. POSTN interacts with a variety of ECM proteins, as shown in Table 1 and Figure 1, possibly anchoring them to the ECM. It may contribute to IVD structure by interacting with different ECM and accessory proteins to maintain their stability and metabolic balance; in turn, these proteins maintain the conditions for the pathophysiological functioning of IVD tissues, and participate in tissue regeneration and fibrosis after injury.

**Table 1:** Multiple proteins interacting with POSTN.

**POSTN Domain**	**ECM Proteins**	**Matricellular Proteins**	**Enzymes**	**Receptors**	**Activity**
EMI	Fibronectin Collagen Laminin γ2	βig-h3	Lysyl oxidase		Binding Interaction
FAS1	Tenascin C	CCN3	BMP-1	Integrins NOTCH1	Binding Interaction
CTD	Proteoglycan				Binding

## POSTN Responds to Mechanical Stress

The magnitude and duration of mechanical stress is positively correlated with the rate of IVD cell apoptosis, which is an important factor leading to IVDD and herniation (4). POSTN, taking its first name from the periosteum and periodontal ligament, is considered sensitive to mechanical stress because the supraosseous periosteum and periodontal ligament can promptly respond to mechanical stress to help tissue regeneration and development (131, 132). A study by Rani et al. confirmed that stress or pressure overload can induce the expression of POSTN, and that IVD overuse and injury are associated with its higher expression (133).

In osteocytes, the degradation of POSTN by cathepsin K plays a central role in the regulation of the biomechanical response in bone tissues and controls cortical bone formation (134). Moreover, Bonnet et al. (135) demonstrated that cortical bone microarchitecture and bending strength related to vertebral degeneration were closely altered in Postn-deficient mice. In the cartilage, POSTN contributed to retaining the shape of a biodegradable polymer scaffold by increasing the mechanical strength of the surrounding fibrous tissues, consisting of a POSTN-mediated collagen structure (136), and enhanced chondrogenesis in a 3D culture embedded within collagen gel; this indicates that conformational changes in collagen induced by POSTN can alter cell adhesion to the substratum and affect signal transduction in chondrocytes (108, 137).

In addition, Stansfield et al. applied a model, as shown in Figure 4B, to investigate the effect of stress on POSTN expression in human NP cells. Following stimulation by mechanical stress for 6 h, the effects of shear stress on the expression of POSTN in degenerative and non-degenerative NP cells were examined by RT-PCR (115). The results revealed that the expression of POSTN and MMP2 was upregulated by mechanical stress, with a significantly higher magnitude of response in human degenerative NP cells compared with non-degenerative cells. This provided an explanation for the susceptibility of degenerative disc cells to stress-induced damage (115). Moreover, Rosselli-Murai et al. (138) found that both POSTN and mammalian target of rapamycin (mTOR) are coordinately upregulated by tension forces during wound healing to induce cell proliferation and migration, indicating that the same signal derived from tendon forces activates the expression of both POSTN and mTOR; in turn, POSTN enhances mTOR signaling.

In conclusion, POSTN expression either promotes or inhibits cellular biological functions to stimulate or hamper irregular collagen fibrillogenesis and ECM organization, in order to maintain or abolish tissue homeostasis in response to mechanical stress, as shown in Figure 4A.

## Pro-Inflammatory Cytokines Inducing POSTN Expression

It is well-known that hypoxia and inflammation are two important characteristics of the environment of IVDD (139). Indeed, high levels of pro-inflammatory cytokines such as tumor necrosis factor (TNF)-α, interleukin (IL)-1 α/β, IL-6, and IL-17 secreted by disc cells induce degeneration of the IVD, which is the major contributor to back/neck and radicular pain (140, 141). These cytokines promote matrix degradation, chemokine production, and changes in cell phenotype (40, 142). Among these, IL-1β and TNF-α are the most important pro-inflammatory cytokines in the inflammatory process because they exert powerful pro-inflammatory activities and can promote the secretion of a variety of pro-inflammatory mediators (143, 144).

Originally, POSTN was identified as a TGF-β-inducible gene as its expression increased in a TGF-β-dose-dependent manner (21). Attur et al. (109) found that TGF-β1 can induce POSTN expression in a time-dependent manner, whereas this is inhibited by IL-1β. POSTN was then found to be induced by various stimuli, including IL-4, IL-13, TNF-β, angiotensin II, connective tissue growth factor 2, BMP-2, and cancer-derived factors (70, 145). Upon stimulation with TNF-α, wild-type and Postn-deficient fibroblasts showed different behaviors. Indeed, the expression of chemokines was significantly upregulated in wild-type fibroblasts, whereas the expression of Ccl2, Ccl7, Cxcl1, and Cxcl2 was markedly downregulated in Postn-deficient fibroblasts (146). This is sufficient and direct evidence of an association between inflammatory cytokines and POSTN expression.

In subsequent studies, POSTN was found to be associated with many chronic inflammatory diseases due to its interaction with inflammatory cytokines. In particular, it was found that POSTN can cooperate with TNF-α or IL-1α derived from epithelial cells or inflammatory cells to activate the NF-κB pathway in fibroblasts, leading to the subsequent induction of various chemokines/inflammatory cytokines including IL-1β, which recruits neutrophils and macrophages and accelerates pulmonary fibrosis (147, 148). Furthermore, the pro-inflammatory cytokines TNF-α and IL-17 work synergistically to enhance the expression of POSTN; this suggests a potential role of POSTN in liver inflammatory injury that eventually leads to fibrosis (149). Not just limited to the liver, the synergistic effect of TNF-α and IL-17 has also been noted in other inflammatory diseases, such as psoriasis, in which it leads to altered keratinocyte differentiation (150). In myocardial ischemia-reperfusion injury (MIRI), POSTN overexpression promotes caspase 1-mediated pyroptosis by activating NLRP3 (151).

**Figure 5:**
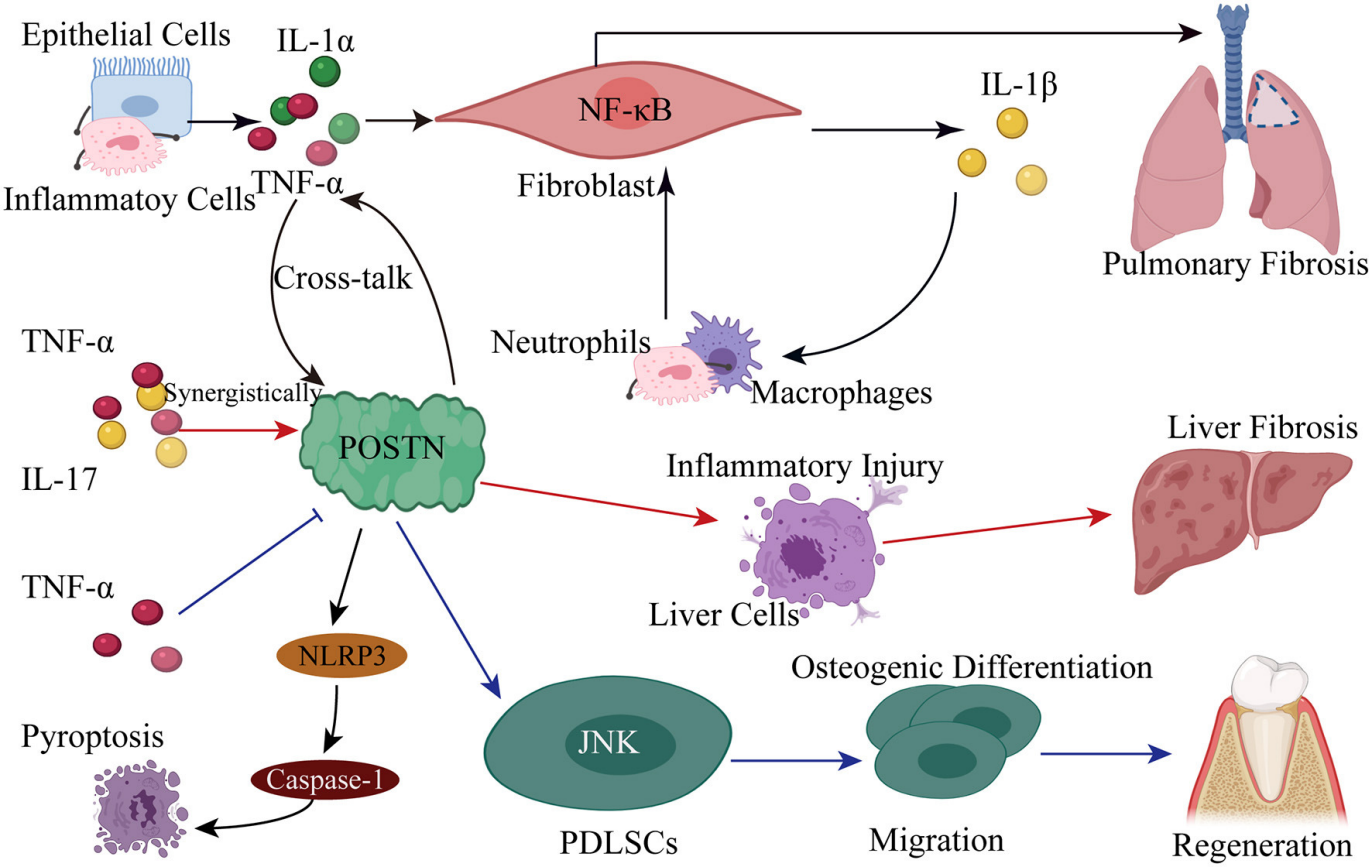
Pro-inflammatory cytokines induce POSTN expression, revealing the underlying mechanism of many chronic inflammatory diseases. (1) POSTN cross-talks with TNF-α or IL-1α derived from epithelial cells or inflammatory cells to induce various chemokines/inflammatory cytokines, including IL-1β, by activating the NF-κB pathway in fibroblasts; this leads to the recruitment of neutrophils and macrophages, which accelerates pulmonary fibrosis. (2) The pro-inflammatory cytokines TNF-α and IL-17 work synergistically to promote the expression of POSTN in order to induce liver inflammatory injury eventually leading to fibrosis. (3) POSTN overexpression promotes caspase 1-mediated pyroptosis by activating NLRP3 in MIRI. (4) POSTN promotes the migration and osteogenic differentiation of PDLSCs by activating the JNK signaling pathway in response to inflammatory stimuli.

Moreover, another study showed that TNF-α inhibited the expression of POSTN at the protein level in PDLSCs (64). POSTN can promote the migration and osteogenic differentiation of PDLSCs by activating the JNK signaling pathway in response to TNF-α challenge. POSTN is also highly expressed during chronic inflammatory diseases such as asthma (152), atopic dermatitis (153), eosinophilic chronic sinusitis/chronic rhinosinusitis with nasal polyps (154), and allergic conjunctivitis (155), and plays important roles in the pathogenesis of these diseases. For instance, POSTN has emerged as a novel biomarker for type 2 inflammation in allergic diseases (148, 154). The interaction between POSTN and inflammatory factors mediates many chronic inflammatory responses; its mechanism of action in this process is shown in Figure 5.

In addition, when Zhao et al. (156) studied the mechanism of *CYP1B1* mutation and congenital glaucoma, they found that *Cyp1b1*^−^/^−^ mice presented ultrastructural irregular collagen distribution in their trabecular meshwork tissue along with increased oxidative stress and decreased levels of POSTN. Interestingly, *Postn*-deficient mice exhibited similar ultrastructural abnormalities in the trabecular meshwork tissue of Cyp1b1^−^/^−^ mice. Therefore, they concluded that the metabolic activity of CYP1B1 contributes to the oxidative homeostasis and the ultrastructural organization and function of TM tissue by regulating the expression of POSTN. In another *in vitro* study, Wu et al. (157) found that oxidative stress-induced periosteal protein is involved in myocardial fibrosis and hypertension. And in a recent study, Liu et al. (158) found that POSTN can inhibit hypoxia-induced oxidative stress and apoptosis in human periodontal ligament fibroblasts via p38 MAPK signaling pathway.

However, to date, there is still no description of the relationship between POSTN and pro-inflammatory cytokines and hypoxia-induced oxidative stress in IVD cells. Although research on how POSTN and pro-inflammatory cytokines lead to IVDD is still lacking, the cross-talk of POSTN with TNF-α and IL-1β, which are the most potent inflammatory cytokines, has been suggested as the underlying mechanism of many chronic inflammatory diseases. In this regard, we can learn from previous studies to outline the possible role of POSTN in the pathophysiology of IVDD.

## POSTN Interacts With Signaling Pathways in IVDD

Current knowledge about POSTN signaling mainly comes from studies aiming to elucidate its role in BMSCs, osteoblasts, chondrocytes, as well as its interaction with inflammatory cytokines and mechanical stimulation. Mechanical stress, chemokines, and changes in ECM composition trigger signaling pathways that induce POSTN expression and subsequent secretion. As a potent modulator of cell–matrix interactions, POSTN has been implicated in the cross-talk between multiple signaling pathways that regulate cell migration, adhesion, and proliferation.

First, the most studied pathway associated with POSTN expression is the TGF-β pathway in osteoblasts cells, in which POSTN acts as a focal contributor of osteogenesis and differentiation in response to mechanical stress (21). Moreover, the binding of POSTN to integrins initiates a cross-talk between integrins and receptor tyrosine kinases, such as EGFR, at the plasma membrane. This crosstalk activates the Akt signaling pathway (159). Akt functions as a cardinal node for the transduction of extracellular and intracellular signals. It is now generally accepted that the PI3K/Akt survival pathway is a central regulator of cell survival and proliferation (160, 161). However, this pathway interacts with the Wnt/β-catenin pathway to induce collagen and proteoglycan degradation in the cartilage (114). POSTN is also capable of activating canonical Wnt/β-catenin signaling in BMSCs and chondrocytes, resulting in induced MMP13 and ADAMTS4 expression to accelerate the pathogenesis of OA, and mediates estrogen-induced osteogenic differentiation of BMSCs in ovariectomized rats to reduce osteoporosis (60, 61, 109).

Not surprisingly, POSTN expression has been shown to activate NF-κB (84, 110, 147, 148), JNK (64), and PERK (82) signaling in BMSCs, osteoblasts, and chondrocytes during development and disease. Finally, mechanical stress activates mTOR signaling to increase the expression of both POSTN and mTOR; in turn, POSTN enhances mTOR signaling (138). In addition, POSTN has been reported to induce MMP2 expression via the avβ3 integrin/ERK pathway in human periodontal ligament cells (162). In a preclinical *in vivo* murine model of human melanoma, POSTN promoted angiogenesis by interacting with integrins avβ3 and avβ5 via its FAS1-2 domain (163). The signaling pathways interacting with POSTN and their effects are shown in Table 2.

**Table 2:** Periostin and its interacting signaling pathways.

**Interacting pathway**	**Cells**	**Function**	**References**
TGF-β pathway	Osteoblasts	Induce differentiation, survival, and apoptosis	(21)
PI3K/Akt pathway	Chondrocytes	Induce collagen and proteoglycan degradation in cartilage	(114)
Wnt/β-catenin pathway	BMSCs	Mediate osteogenic differentiation of BMSCs to reduce osteoporosis	(60, 61)
	Chondrocytes	Induce MMP13 and ADAMTS4 expressionAccelerate the pathogenesis of OA	(109)
NF-κB pathway	Chondrocytes	Upregulation of inflammatory cytokines and MMPs	(110)
	Osteoblasts	Induce expression of RUNX2 and effects on osteoblast differentiation	(84)
	Fibroblasts	Accelerate fibrosis	(147, 148)
mTOR pathway	Epithelial cells	Mediate mitogenesis and migration of cells	(138)
JNK pathway	PDLSCs	Promote migration and osteogenic differentiation	(64)
PERK pathway	Osteoblasts	Inhibit melatonin-induced cell apoptosis	(82)
**Other pathways**			
FAS1-integrins pathway	Melanoma cells	Promote angiogenesis	(163)
Integrin/ERK pathway	Periodontal ligament cells	Induce MMP2 expression	(162)

## Potential of POSTN as a Biomarker

POSTN has been intensively studied as a biomarker for inflammatory and allergic diseases. In addition to its role in respiratory diseases such as asthma and allergic pneumonia (147, 148), it was found that plasma and SF POSTN levels were positively correlated with the radiographic severity of knee OA, and POSTN expression was increased in synoviocytes and SF from OA patients (106, 164). Moreover, a clinical study directly showed that serum POSTN (sPOSTN) levels were associated with the prevalence and risk of development and progression of knee OA in women (165). Additionally, POSTN variants are also associated with the course and severity of juvenile idiopathic arthritis, according to the literature (166).

Significantly, an increasing amount of information is available about the role of POSTN in fractures and osteoporosis, which is the most common age-related bone disease characterized by bone resorption (50). A longitudinal case-control study of women over 70 years of age with acute osteoporotic hip fractures suggested that high levels of sPOSTN are associated with bone density loss, because women with hip fractures exhibited higher sPOSTN levels (167). Furthermore, high sPOSTN levels were regarded as independent predictors of femoral neck bone mineral density in older women with acute hip fractures (168). In addition, sPOSTN levels were higher in ankylosing spondylitis patients with higher disease activity and higher systemic inflammation and were independently associated with C-reactive protein levels (169). This study has shown for the first time a positive association among POSTN expression, inflammatory markers, and disease activity. Finally, sPOSTN levels are even increased in fractures during the early healing phase, possibly implying that POSTN plays a role in bone repair (167). When vertebral and non-vertebral fractures were analyzed separately, it was found that POSTN levels were associated only with the latter fracture type, in agreement with the predominant role of POSTN in cortical bone metabolism established by a study on the role of sPOSTN in postmenopausal women (87, 168).

Therefore, in view of the characteristics of POSTN in clinical diseases, an increasing number of scholars have proposed POSTN as a biomarker for predicting the severity of OA and diagnosing osteoporosis and the outcome of fracture repair (170, 171).

## Conclusion and Perspectives

In this article, we described the roles of POSTN in BMSCs, osteoblasts, osteocytes, and IVD cells, as well as its interactions with inflammatory cytokines and mechanical stimulation, and the potential of POSTN in acting as a biomarker of some diseases. POSTN was originally identified in an osteoblastic cell line (20) and its expression was found to be prominent in the bone periosteum, indicating its involvement in bone tissue development and function (21). POSTN plays a critical role in regulating bone microarchitecture, strength, and mass by promoting BMSC and osteoblast differentiation and survival, and regulating collagen fibrillogenesis and ECM assembly (62, 122). On the other hand, increased POSTN expression could contribute to bone mass protection in hyperparathyroidism (91). Furthermore, POSTN is present in disc cells and can promote the degradation of cartilage ECM and induce chondrocyte apoptosis (109, 110). Finally, the cross-talk of POSTN with pro-inflammatory cytokines reveals the underlying mechanism of many chronic inflammatory diseases (40, 142, 149).

Although the study of POSTN has developed dramatically in the past several years, several problems connected to its role in IVDD have remained unresolved: (a) POSTN expression has been officially reported in NP, AF, and osteoclasts. However, almost nothing is known about the mechanism by which POSTN interacts with these cell types to mediate degeneration. (b) Increased POSTN expression was observed in both SF and serum of patients with higher disease activity and higher systemic inflammation, and thus this gene can potentially be used as a biomarker. Therefore, there is a relationship between sPOSTN levels and the Pfirrmann grade of spinal degeneration. In brief, sPOSTN can also be used as a biomarker for spinal degenerative diseases. (c) Many studies have suggested that inflammation is an important factor that leads to herniation, hypoxia, and IVDD. Therefore, it is important to investigate the underlying mechanism by which POSTN responds to inflammatory cytokines during IVDD. (d) POSTN is sensitive to mechanical stress. POSTN expression is increased under mechanical stress as well as under overstimulation and injury. In the process of IVDD, the apoptosis of NP cells caused by external mechanical stress is the most important; therefore, the role of POSTN in the process of mechanical stress-induced apoptosis of NP cells needs further study. However, studies in these areas have been lacking so far. We hope that in the near future novel studies will provide an improved and broader understanding of POSTN activity in the pathogenesis of spinal degenerative diseases, yielding useful diagnostic or therapeutic agents to counteract them.

## Author Contributions

DZ, ZW, and WZ contributed equally to the writing of this article, conceived and designed the study, and drafted the manuscript. YW, ML, and GZ referenced manuscript analysis. XG and XK edited and revised the manuscript. All authors contributed to the article and approved the submitted version.

## Conflict of Interest

The authors declare that the research was conducted in the absence of any commercial or financial relationships that could be construed as a potential conflict of interest.

## Publisher's Note

All claims expressed in this article are solely those of the authors and do not necessarily represent those of their affiliated organizations, or those of the publisher, the editors and the reviewers. Any product that may be evaluated in this article, or claim that may be made by its manufacturer, is not guaranteed or endorsed by the publisher.

## Abbreviations

AF: Fibrous annulus
BMSCs: Bone-marrow-derived mesenchymal stem cells
CTD: C-terminal end
ECM: Extracellular matrix
HSCs: Hematopoietic stem cells
IVDD: Intervertebral discs degeneration
IVD: Intervertebral discs
MMP: Matrix metalloproteinase
NP: Nucles pulposus
OA: Osteoarthritis
POSTN: Periostin.
